# EXPERIENCES OF PARTICIPATION IN CARDIORESPIRATORY TRAINING AMONG PEOPLE WITH POST-STROKE FATIGUE: A QUALITATIVE STUDY

**DOI:** 10.2340/jrm.v57.42282

**Published:** 2025-04-16

**Authors:** Maria SVEDJEBRANT, Anna BRÅNDAL, Ylva NILSAGÅRD

**Affiliations:** 1 Centre for Research and Development, Region Gävleborg/Uppsala University, Uppsala; 2 Faculty of Medicine and Health, Örebro University, Örebro; 3 Department of Public Health and Clinical Medicine and Department of Community Medicine and Rehabilitation, Physiotherapy, Umeå University, Umeå; 4 University Healthcare Research Centre, Region Örebro, Sweden

**Keywords:** cerebrovascular disorders, post-stroke fatigue, cardiorespiratory training, qualitative study, physiotherapy

## Abstract

**Objective:**

To explore the experiences of 8-week thrice-weekly supervised intense cardiorespiratory interval training at home in people with post-stroke fatigue.

**Design:**

Qualitative, using semi-structured individual interviews.

**Methods:**

Eleven people with post-stroke fatigue were recruited from a study investigating the effects of supervised intense cardiorespiratory interval training. The interviews were conducted 1–2 weeks after the intervention period and analysed inductively with qualitative content analysis.

**Results:**

The theme “Supervised cardiorespiratory interval training at home was perceived as feasible and safe, reducing fatigue and contributing to enhanced self-efficacy in both exercise and everyday life” was based on 4 main categories: “Experiences of performing the intervention and testing, “Perceived impact of the intervention on fatigue”, “Perceived impact of the intervention on self-efficacy”, and “The reinforcing experiences of exercise transfer to everyday life”. The informants reported that participating in the intervention made them feel physically and mentally stronger and no longer restricted by a lack of confidence in performing activities that increased their heart rate.

**Conclusions:**

Participating in supervised intense cardiorespiratory interval training provided a model on how to train, which was transferable to other contexts in everyday life. The training improved exercise self-efficacy and gave the informants the confidence to challenge themselves in everyday life.

## LAY ABSTRACT

Post-stroke fatigue is a common symptom after a stroke, but the mechanisms behind it are not well understood. Many people with stroke have low physical capacity, and post-stroke fatigue can be a barrier to training. Despite the potential benefits of intense training, we lack insight into how people with post-stroke fatigue experience such training. This study aimed to explore how people with post-stroke fatigue experience intense cardiorespiratory interval training at home. We interviewed 11 individuals with post-stroke fatigue to gather their experiences. The results showed that participating in supervised cardiorespiratory interval training provided participants with a model for how to exercise, which they could apply to other areas of their daily lives. It boosted their confidence and helped them to challenge themselves in everyday activities. Support should be offered in managing intense training after stroke, and those with post-stroke fatigue should not be excluded.

Stroke is the second leading cause of death and disability globally and a major contributor to disability-adjusted life years in those with neurological disorders (1). Post-stroke fatigue (PSF) is a common functional limitation with a pooled prevalence of 47% (95% CI 43–50%) (2). Stroke survivors rank understanding fatigue and understanding how to reduce fatigue as high priorities for research (3). PSF affects the patient’s level of activity and quality of life and often persists over a long period of time (4). National guidelines recommend that people with stroke undergo targeted secondary prevention to support long-term management of risk factors for stroke, which also includes early initiation of cardiorespiratory fitness training (5). Increased cardiorespiratory fitness could be beneficial for a range of common post-stroke problems, such as disability, fatigue, and increased energy expenditure due to hemiplegic gait (6).

Stroke rehabilitation often consists of low-intensity aerobic activities and is associated with a high degree of sedentary time (7). Barriers to adequate aerobic training during routine stroke rehabilitation include lack of equipment, time, and staff, as well as insufficient knowledge and skills in the safe prescription and implementation of aerobic training (8). Instead, moderate physical exercise (e.g., walking) and balanced activity are often applied in managing PSF (9).

PSF can be a barrier to participation in physical activity (PA) promotion programmes (10) and maintaining a sufficient PA level after stroke rehabilitation (11). Even people with mild stroke report that PSF is one of the main obstacles to PA, and inactivity can lead to increased fatigue, which further interferes with the ability to be physically active (10). Belief in one’s capacity to perform activities is important in stroke recovery (12), and exercise self-efficacy is directly associated with quality of life in people with stroke (13).

People with stroke have lower physical capacity compared with unimpaired individuals (14) and spend more time in sedentary behaviour (15). They also perform activities at a significantly higher relative aerobic load compared with peers without impairments (16). PA interventions could target multiple potential biological mechanisms present in PSF, including the suppression of inflammation (17). Improved physical fitness may protect against PSF (18).

Cardiorespiratory training may have the potential to relive PSF and improve functional capacity and quality of life but is an underutilized therapeutic intervention. To properly advise people with stroke in managing fatigue, caregivers need to have a better understanding of PSF (19). Although PSF is common and negatively influences recovery, few studies have evaluated targeted fatigue interventions, and there are currently no treatment guidelines (20). High-intensity interval training is superior to moderate-intensity continuous training in improving cardiorespiratory fitness in patients with cardiovascular disease (21) and is feasible and safe in patients with lacunar and minor stroke (22).

Experiences of participating in cardiac rehabilitation after stroke have been described (23, 24), but descriptions of training interventions in people with PSF are lacking. This study therefore aimed to explore the experiences of people with PSF when participating in an 8-week thrice-weekly supervised cardiorespiratory interval intense training programme (S-CITP).

## METHODS AND MATERIALS

### Design

A qualitive semi-structured interview design was used (25).

### Study context

The informants were recruited from a 2-centre randomized controlled trial (RCT), not yet published (Clinicaltrials.gov NCT03458884; Swedish Ethical Review Authority Umeå, 2015-420-3M and Gävle 2019-03207). The RCT started at Umeå University Hospital in May 2018 and was expanded to Gävle Hospital in November 2019.

People with PSF were randomized to control or a thrice-weekly cardiorespiratory interval training over 8 weeks as a complement to Early Supported Discharge (ESD) services. The ESD service provides support and rehabilitation for patients in their own environment, and the rehabilitation is often focused on activities of daily living and mobility, including fatigue management.

Participation in the RCT included performing an incremental cardiopulmonary exercise test on an ergometer cycle before and after the intervention. The cardiorespiratory interval training (S-CITP) consisted of ergometer cycling performed at home and supervised by a physiotherapist specialized in neurology. The S-CITP included a warm-up followed by 4 x 4-min intervals, which were interrupted by 3 min of active recovery and 5 min of cool down after the interval session. Participants wore a heart rate monitor during the training and rated perceived exertion and were encouraged to reach a level of physical effort of 15–16 (corresponds to a “hard” level of exertion) on the Borg Rating of Perceived Exertion Scale (26) every minute during the 4-min intervals.

### Participants and sampling

Starting in November 2019 and through to December 2023, 12 people with PSF were included and randomized to the intervention group. These individuals were invited in person by their treating physiotherapist to participate in the present study at the end of the training period. Both written and verbal information were provided, and written consent was required. All but 1 agreed to participate. Informants were not asked to provide a reason for declining participation.

The informants were medically stable adults living independently in Umeå (*n* = 4) or in Gävle and the surrounding area (*n* = 7). When entering the RCT, they were able to walk unassisted, had no severe cognitive dysfunction (Montreal Cognitive Assessment ≥ 26/30 points ) (27), and rated PSF ≥ 28/50 (28–40) points according to the Swedish Fatigue Assessment Scale (S-FAS) (28). Three participants were single, living alone, and 8 lived with their spouses. Informant characteristics are presented in Table I.

**Table I:** Characteristics of the study informants

Informant	Male/female	Age (years)	Time since stroke onset (months)	Modified Rankin scale (0–6)
1	Male	51	2	2
2	Female	32	3	2
3	Female	68	5	2
4	Female	47	5	2
5	Male	68	4	1
6	Male	73	5	2
7	Female	56	5	2
8	Female	65	5	2
9	Female	61	4	2
10	Male	53	3	1
11	Female	68	3	1

### Data collection

Semi-structured individual interviews were conducted by the first author (MS) between 1 and 2 weeks after the intervention period. All (female) authors are registered physiotherapists (RPT) with specialist qualifications in the field of neurology and extensive clinical experience in stroke rehabilitation. MS has completed a postgraduate course in qualitative content analysis and has received supervision in the method from YN (PhD, Associate Professor) and AB (PhD), who have published articles using this method. MS and AB were treating physiotherapists in the RCT at the respective site (Gävle Hospital *n* = 7, Umeå University Hospital *n* = 4). The participants were aware that the interviewer (MS) was an RPT interested in PSF and a PhD student. YN was not actively involved in the treatment or data collection.

The following starting points were applied when producing the interview guide: open-ended questions should be asked covering pre- and post-treatment measurements of cardiorespiratory function and the intervention period. Of specific interest was capturing experiences of exercise intensity, exercising at home and under supervision of an RPT, positive and negative experiences regarding the exercise mode, whether PSF was changed after the training period, and whether participants experienced any changes in their everyday life (see interview guide, Appendix S1). To avoid interviewer bias, the interviews were conducted before the RCT results were analysed.

In the first interview, the informant was initially asked to talk generally about their stroke. The relevance of the interview content in relation to the study aim was discussed between the co-authors. To address the informants’ fatigue and strengthen the focus on the research questions, this topic was asked about at the end of the subsequent interviews. As fatigue is a broad concept, the interviewer stressed that the interview questions concerned new fatigue arising after the stroke. Prompting questions encouraged the informants to elaborate on the themes. The informants were encouraged to express both negative and positive experiences. The interviewer gave the informants the opportunity to provide complementary information at the end of each interview.

In Gävle and its surrounding area, in-person interviews were conducted at the informants’ home. Due to pandemic travel restrictions, interviews in Umeå were conducted online. In both cases, only the interviewer and the informant were present. All interviews were audio-recorded and transcribed verbatim by MS. The first interview lasted 53 mins, and the following between 20 and 38 min.

### Data analysis

Data were analysed with qualitative inductive content analysis, as suggested by Graneheim and Lundman, to capture the variation in experiences (25).

To facilitate the structuring and qualitative analysis of data, the first author (MS) used the software program NVivo (release 1.6.1; https://lumivero.com/products/nvivo/). To create an overall picture, ensure that nuances were captured, and that no significant information was overlooked, all 3 authors read the 11 interview transcripts several times during the process in the following steps:

1. In the first 3 interviews, 2 of the authors (MS, AB) met to identify meaning units directly related to the research question. Meaning units were first identified separately and then together to identify potential differences and similarities.
2. Thereafter, the third author (YN) identified meaning units in 2 of these interviews, and all authors discussed potential differences and similarities.
3. For the following interviews, MS identified and condensed the meaning units and coded them. The codes were subsequently implemented using NVivo.
4. All authors scrutinized the codes and independently suggested labels for sub-categories, then analysed the codes for grouping into the sub-categories.
5. All authors discussed the sub-categories, which resulted in refinements of the labels by MS.
6. Further discussions took place between all authors to identify main categories into which the sub-categories were clustered.
7. A descriptive theme that illustrated the manifest content connecting all categories was formulated in consensus among all authors.
8. Lastly, all authors reinforced the results by selecting quotes.

All interviews were analysed and discussed several times during this process with all authors until consensus was reached. The informants were not asked to provide feedback on the findings. The quotes were translated, and language edited when the analysis was complete. The study is reported according to the Consolidated Criteria for Reporting Qualitative Studies (COREQ) guide (29). Examples of meaning units, sub-categories, and categories are illustrated in a coding tree (see Table II).

**Table II:** A coding tree to illustrate the analysis process

Meaning units	Codes	Subcategory	Main category
“I didn’t know what I could train. Now I have seen it with a physiotherapist first, and that was reassurance, that there was someone who encouraged me, saying this is the right level, and that was really good, because I couldn’t have done it myself” (Informant 8)” “It’s on my mind, being afraid of another stroke or another heart attack. But that was maybe at the first and second session, since then it has been fine to achieve an increase in heart rate” (Informant 4)	Training model Important that someone is present to dare	Supervised training	Experiences of performing the intervention and testing
“I feel better, have more energy, and I’m starting to recognise myself as I used to be” (Informant 9) “Now it’s about an hour of rest, even if I’ve done something strenuous, I recover much faster” (Informant 2)	Exercise gives energy Need for rest	Time for recuperation	Perceived impact of the intervention on fatigue
“Now when my heart rate goes up, ‘‘m not afraid anymore” (Informant 5) “Yes, but it’s just in my head, oh how hard it is. But once you start, it’s not that bad. It feels good that you can handle it. You can handle it and feel good from it” Informant 9)	How the body reacts to exercise I have learned more about how my fatigue works	Body awareness	Perceived impact of the intervention on self-efficacy
“Since I have gotten better fitness, I can handle things much better, like cooking and going to the store to shop and all that” (Informant 5) “It has been good for my mind. I start thinking more clearly and better, and I can concentrate better” (Informant 6)	Impact on activity level Effect on concentration	Impact on daily activities	The reinforcing experiences of exercise transfer to everyday life

### Ethical considerations

The Swedish Ethical Review Authority approved the study (Dnr 2019–04939). The study followed the ethical requirements of the Declaration of Helsinki. Informed written consent was obtained.

## RESULTS

Four main categories and 11 subcategories were identified in the analysis of the informants’ experiences of S-CITP (Table III). The essence of the content was formulated in a manifest level theme (25): “Supervised cardiorespiratory interval training at home was perceived as feasible and safe, reducing fatigue and contributing to enhanced self-efficacy in both exercise and everyday life”.

**Table III:** Sub-categories, categories, and theme illustrating the experiences of thrice-weekly physiotherapist supervised cardiorespiratory interval training over 8 weeks in people with post-stroke fatigue

Supervised cardiorespiratory interval training at home was perceived as feasible and safe, reducing fatigue and contributing to enhanced self-efficacy in both exercise and everyday life										
Experiences of performing the intervention and testing					Perceived impact of the intervention on fatigue		Perceived impact of the intervention on self-efficacy		The reinforcing experiences of exercise transfer to everyday life	
Performing training at home	Cycling as training	Performing the cardiopulmonary exercise test	Supervised training	Scheduled training	Time for recuperation	Awareness of post-stroke fatigue	Body awareness	Confidence in performing physical activity	Next-of-kin insight	Impact on daily activities

After discharge from hospital, many informants reported that it was difficult to “live as usual”, even if they had only mild physical impairments. PSF was described as a barrier to increased PA, and they feared that strenuous activities could cause harm. Participation in S-CITP was described as something that made them both physically and mentally stronger. Training also gave them more insight into PSF and more confidence in performing training, an insight that they could translate to other activities in daily life. They reported having more confidence and energy and were able to challenge themselves both physically and socially, which was perceived to make daily activities easier.

### Experiences of performing the intervention and testing

The informants revealed different experiences, but there were also similarities in their stories. Several informants described that it was advantageous that the S-CITP was performed at home and that it was planned, structured, and supervised. They stated that it would have been too energy consuming to travel to a training facility and that it would have increased their dependence on their next of kin. Overall, the informants stated that being able to train at home made the experience less stressful.

*Yes, but it’s great. It’s superb. It has been very nice to not have to go away from home. It has been very nice, and it also makes it less stressful, not to be dependent on somebody giving me a ride, since I’m not allowed to drive. (Informant 3)*

The informants consistently stated that the frequency and duration of the training was adequate. The fact that everything was organized and scheduled was generally seen as a positive; however, 1 informant mentioned that having a scheduled date and time could be difficult due to the lack of flexibility. The actual performance of the incremental cardiopulmonary exercise test was perceived as positive and engaging, but the need to wear a mask that measures oxygen was seen as a source of mild discomfort.

It was crucial for the informants that the training was supervised, as several expressed a fear of pushing themselves physically. Supervision by a physiotherapist made them feel safe during training. Supervision was also a key factor in enabling informants to reach the intended intensity. They said that they would not have reached these intensity levels on their own. It was also important to be encouraged to train regardless of PSF.

*It’s on my mind, being afraid of another stroke or another heart attack, but that was maybe at the first and second session, since then it has been fine to achieve an increase in heart rate. (Informant 4)*

*It felt safe to be able to make the kind of effort I did on the bike, to do it under supervision, otherwise … maybe I would still be on the couch. Being afraid of doing anything at all due to the onset of fatigue. (Informant 1)*

There were different opinions regarding training on the ergometer cycle. Some stated that it was too repetitive, while others stated that it was a stable form of exercise that did not require strong balance.

### Perceived impact of the intervention on fatigue

Preventing PSF from occurring was not possible, but many informants mentioned that they now needed less time for recuperation. The shorter recuperation time was considered to have a positive effect on their fatigue.

*It feels like I recover better when I am tired. I can’t explain it in any other way. It takes longer before I feel a lack of strength, and then I recover faster. (Informant 11)*

The informants stated that their fatigue decreased but was still present, especially in environments with many visual distractions and sounds and in social situations. None of the informants reported increased PSF after the training session. In fact, informants reported feeling more energetic. They also mentioned that participating in the training was perceived to have a positive impact on their general activity level.

*It gives me so much energy. It’s just like the endorphins are dancing in my body, and you feel healthy and good from the training. (Informant 2)*

*I have become much happier, and I have got my energy back. And so I believe it helped my thought process, so I don’t have to think and worry about the same things over and over. I escape mentally during training. It feels good. (Informant 2)*

### Perceived impact of the intervention on self-efficacy

Informants stated that the ability to train again increased their awareness of their body and changed the way they experienced fatigue. Participating in the S-CITP was perceived to build confidence, a kind of confidence that was transferable to other physical activities.

*So, nowadays, I know how it feels in my body if I push myself too much, and that helps me while training on my own. I feel like I have taken control over myself again. (Informant 10)*

S-CITP was also perceived to impact the informants’ general well-being in terms of mood and feelings of physical confinement due to the fear of exhaustion. Several informants described that they did not have enough confidence to perform more physically demanding activities before participating in the S-CITP.

*Before [the S-CITP], I was more uncertain. I didn’t know what I physically could do. But this training has given me confidence. I feel that this type of physical activity is good for me. It’s not something that makes it worse. It’s rather the opposite. It makes me feel safe when training at this level of exertion. (Informant 10)*

### The reinforcing experiences of exercise transfer to everyday life

The informants stated that PSF limited their daily activities. Activities they had performed before their stroke (e.g., managing different social contexts) became more difficult. They described that S-CITP had a positive impact on stress management in daily activities. They also felt both physically stronger and “clearer in the head”, which, for example, increased their ability to participate in social activities. Everyday chores that were previously done out of necessity were now enjoyable. Participation in the S-CITP was also perceived to make it easier for them to handle complex activities like baking and cooking.

*I think I have a better way of handling the situation. I have more endurance today. I participate in social situations today. I can participate in activities in a different way than before. (Informant 10)*

The informants felt that life became more like before the stroke, even though some PSF remained. By pushing themselves during training, they were able to test their physical limits, which made them more confident in their abilities in everyday life. This was described as a feeling that they could continue to live their lives in a good way.

*I am not afraid to do more and more. I’ve started to ride the bike. I use a kick sled. I decided to start living as usual. (Informant 11)*

It was revealed that it was important for the informants that their spouses were involved, because several informants had family members who restricted their activities following hospital discharge. They described that their spouses were afraid that the level of intensity was not beneficial after a stroke. At-home training eased that concern and enabled spouses to feel less concerned about their partner performing activities in everyday life that increase the heart rate.

*When he realized that I could ride the bike like I do, he has understood that I can do this (training). So, he has realized that I can manage to do things. He doesn’t have to protect me. I can handle things on my own. And I don’t have to defend myself, since he understands that I can, that I have the strength. He sees, he hears, and he understands. (Informant 11)*

## DISCUSSION

Overall, the physiotherapist-supervised thrice-weekly cardiorespiratory interval training was described as a positive experience, making the informants feel physically and mentally stronger and allowing them to live their lives in a way that was more like before the stroke. They were no longer restricted by fear or a lack of confidence in performing activities that increased their heart rate. They felt less restricted by PSF, although some symptoms persisted. Their next of kin no longer held them back from strenuous activities. It is likely that the present findings, where informants reported feeling more energetic and less fatigued, are related to better cardiorespiratory fitness.

The informants expressed that before participating in the intervention, they feared that fatigue would worsen when they were training, but that this was not the case. It has been reported that fatigue is not exacerbated by exercise in people a year or longer post-stroke (30). These findings are important for clinical management of PSF as physiotherapists may consider PSF to be a barrier to aerobic training immediately following a stroke (31). Fatigue is one of the main reported barriers to exercise for people with PSF (10), and a lack of confidence can create a sedentary cycle that may be difficult to break without support. Individuals with stroke experience a high relative aerobic load during cyclic daily-life activities, despite adopting a slower pace of movement compared with peers with no impairments (16).

The informants stated that participation in the S-CITP “cleared their head”. For example, they reported that after the intervention period, they were more able to main attention, which facilitated participation in different social situations.

The informants also expressed that one important factor was that the S-CITP took place in their homes. Distance to training premises and the need for transport services have been reported as barriers to participation in training post stroke (10). PSF often makes it difficult for individuals to function in busy environments, which poses challenges to rehabilitation in regular training facilities. Training at home also provides spouses with important insight into their partner’s capacity to exercise. This shows that it is crucial that family members receive information concerning the importance of exercise and that strenuous activities are beneficial after a stroke. Family members can constitute a barrier to PA and cardiorespiratory training after stroke (32).

The informants valued the presence of a physiotherapist during the training and this was considered necessary for reaching the required intensity during cardiorespiratory training. This is in line with a previous study, which showed that supervised exercise was a top priority for people with PSF (33).

In clinical practice, activity-specific balance training and moderate-intensity physical training are commonly used in PSF management. Time of hospital discharge has been identified as a window of opportunity, that is, a period where motivation for engagement in training is high, and a “teachable moment” where there is greater receptiveness to education and lifestyle change (34). This early period presents unique challenges but also opportunities to foster positive beliefs, change patient perspectives, and reduce long-term sedentary behaviours (35). Based on our clinical experience, people with PSF are not usually offered cardiorespiratory training at this high level of intensity.

Considering all of the pathways through which cardiorespiratory training can reduce fatigue, including multiple potential biological mechanisms present in PSF (17) and its positive effect on mechanisms of neuroplasticity (36), it is surprising that cardiorespiratory training receives so little attention in the early phase of stroke rehabilitation. As is the case with motor recovery studies, most training trials have started in the chronic phase, 2 or more years post stroke (37). Delaying motor rehabilitation until the chronic phase makes it progressively more difficult to achieve significant recovery (38). The fact that training can reduce fatigue in stroke recovery has been reported (39), but the present study differs in the intensity of exercise and time after stroke.

People with stroke rate the importance of participating in cardiorespiratory training higher after a stroke (40). The informants in this study described a desire to be able to exercise, but they did not know how to do so before participating in the intervention. A lack of information on how to perform cardiorespiratory training (40) and the fear of another stroke (41) have been reported as barriers, which is in line with the findings of the present study. It was clear that the informants did not have sufficient information or confidence on how to exercise. Participating in the S-CITP provided a model on how to train, which was also transferable to other contexts in daily living. Importantly, the S-CITP gave informants the confidence to challenge themselves in daily activities, including training, thus improving exercise self-efficacy. It is important that people with PSF do not become resigned to their condition and resistant to training because they perceive it as potentially harmful. Supervised cardiorespiratory training in people with PSF may prove to be an important bridge, leading to independent self-management of PA and higher intensity PA. Standard rehabilitation may not reach the aerobic intensity required to elicit aerobic benefit, and people with PSF may be excluded due to insufficient knowledge.

### Strengths and limitations

Using a qualitative design with individual interviews allowed for follow-up questions, which deepened the narratives from the informants. The interviews were rich in data and maintained a strong focus on the research question. Despite the limited number of informants, the findings from the present study may be valid for post-stroke patients who experience fatigue and have an interest in participating in training. The sample included both men and women of different ages, and all informants had significant PSF.

The interviewer had knowledge of stroke rehabilitation and insight into the intervention. This can be seen as a strength as well as a weakness. Self-reflection is an essential part of qualitative research, and the researchers’ pre-understandings must be considered, as this may affect data collection and the analysis process. While pre-understanding can create opportunities to discover new knowledge and a deeper understanding of what is being studied (42), the informants may have been reluctant to express negative aspects related to participation when the interviewer supervised the training in Gävle and its surrounding area. The interviewer encouraged the informants to be forthcoming with any negative feedback. When analysing and comparing the interviews, there were no differences between the 2 centres regarding negative feedback.

There is always a risk of inconsistency in data collection with inclusion over a longer period. However, adherence to the interview guide during the process ensured that content was similar during the interviews, despite the long data collection period. To strengthen the trustworthiness of the results, all authors met regularly and discussed content and analysis during all stages of the process. The purpose was to maintain objectivity and consensus in the analysis and to add different perspectives and interpretive possibilities. The dependability of the analysis process is important to consider as each author had their own pre-understanding and interpretive repertoire to consider. To strengthen the dependability of the study, all authors took an active role in the analysis process until consensus was reached. The interpretation of the results was further reinforced by quotations to ensure the transparency of the analysis.

The decision to explore a qualitative aspect of the S-CITP was made some time after the RCT was started. Thus, participants who had ended their training period before that decision could not be invited to participate. However, the sample size in qualitative studies can be guided by information power. The present study had a narrow study aim. All authors read all transcripts to ensure that the quality of the dialogue was sufficiently strong, included a consistent focus on the research question, and that the descriptions were rich. Because no new data emerged from the last 2 interviews it was considered that enough data were collected to answer the research aim.

A strength of the study is that the interviews were conducted close in time to the end of the intervention to minimize recall bias. The results have limited generalizability due to the positive selection of informants, as only informants who completed the S-CITP were interviewed. It is a limitation that we do not have information on the informants’ prior exercise experience, as there is reason to believe that those who expressed an interest in participating in a training intervention would also have a more positive view of training in general.

We concluded that participating in supervised intense supervised cardiorespiratory interval training provided a model on how to train, which was transferable to other contexts in everyday life. The training improved exercise self-efficacy and gave the informants the confidence to challenge themselves in everyday life.

### Implications for practice

This study contributes new knowledge on the importance of offering cardiorespiratory training to people with PSF. Based on our clinical experience, this intensity level of exercise has not commonly been recommended this early after stroke onset. Cardiorespiratory training is not only a crucial component in secondary prevention, but also in managing PSF. Further research is needed to increase the knowledge of cardiorespiratory training and how it can be embedded in PSF management strategies, as has been successfully done for survivors of cardiac events.

## Supplementary Material

EXPERIENCES OF PARTICIPATION IN CARDIORESPIRATORY TRAINING AMONG PEOPLE WITH POST-STROKE FATIGUE: A QUALITATIVE STUDY
