# Supplementary material for: EXPERIENCES OF PARTICIPATION IN CARDIORESPIRATORY TRAINING AMONG PEOPLE WITH POST-STROKE FATIGUE: A QUALITATIVE STUDY
Source: J Rehabil Med. 2025 Apr 16;57:42282. doi: 10.2340/jrm.v57.42282 (PMC12016664; doi:10.2340/jrm.v57.42282)
Supplement: EXPERIENCES OF PARTICIPATION IN CARDIORESPIRATORY TRAINING AMONG PEOPLE WITH POST-STROKE FATIGUE: A QUALITATIVE STUDY [file JRM-57-42282-s1.pdf]

Supplementary material has been published as submitted. It has not been copyedited, or typeset by Journal of Rehabilitation Medicine

Appendix S1

Interview guide

Themes

Post-stroke fatigue

- Could you explain how you experience fatigue?
- Has your fatigue altered over time?
- How did your fatigue feel after training?
- After training, have you been able to perform things during the day?
- How was it to recuperate after training?
- Has the training had any impact on your daily activities?
- Did the training have any impact on your fatigue?

Cycling

- What was your experience of the cardiorespiratory training?
- How did it feel to cycle intervals?
- How did you experience the training period?
- How has it been to train at home?

Exercise test

- Please describe your experience with the cardiopulmonary exercise test

Background

- Could you describe your stroke and its effects on you?
- In your free time, what do you prefer to do?
- Can you tell us about what a typical day looks like?
- How physically active are you during the day?
- Can you describe any strategies you have to deal with your fatigue?

Completion
